# Process-time Optimization of Vacuum Degassing Using a Genetic Alloy Design Approach

**DOI:** 10.3390/ma7127997

**Published:** 2014-12-10

**Authors:** David Dilner, Qi Lu, Huahai Mao, Wei Xu, Sybrand van der Zwaag, Malin Selleby

**Affiliations:** 1 Materials Science and Engineering, KTH Royal Institute of Technology, Brinellvägen 23, SE-100 44 Stockholm, Sweden; E-Mails: huahai@kth.se (H.M.); malin@kth.se (M.S.); 2 Novel Aerospace Materials group, Faculty of Aerospace Engineering, Delft University of Technology, Kluyverweg 1, 2629 HS Delft, The Netherlands; E-Mails: q.lu@tudelft.nl (Q.L.); w.xu@tudelft.nl (W.X.); s.vanderzwaag@tudelft.nl (S.Z.); 3 Thermo-Calc Software AB, Norra Stationsgatan 93, SE-113 64 Stockholm, Sweden; 4 ArcelorMittal Globle R&D Gent, J.F. Kennedylaan 3, 9060 Zelzate, Belgium

**Keywords:** genetic algorithm, computational thermodynamics, vacuum degassing, materials by design, steelmaking

## Abstract

This paper demonstrates the use of a new model consisting of a genetic algorithm in combination with thermodynamic calculations and analytical process models to minimize the processing time during a vacuum degassing treatment of liquid steel. The model sets multiple simultaneous targets for final S, N, O, Si and Al levels and uses the total slag mass, the slag composition, the steel composition and the start temperature as optimization variables. The predicted optimal conditions agree well with industrial practice. For those conditions leading to the shortest process time the target compositions for S, N and O are reached almost simultaneously.

## 1. Introduction

Several approaches to predict materials properties have been developed over the past decades. Among those the CALPHAD-method [1,2], an approach for compuational thermodynamics, is one of the most widely applied. These models have often been used to confirm and complement experimental work. Currently there is an increased interest to use modeling as the main tool for the development of new materials. The materials by design approach has successfully been used at Northwestern University to develop high strength steels [3,4] for aerospace applications. In their study a suite of coupled models are combined with the specialist intervention to find optimal compositions and heat treatment temperatures. At TU Delft a genetic algorithm (GA) approach not requiring specialist intervention was developed to search a multi-parameter search domain on the basis of thermodynamic models, clear quantitative constraints and a single quantitative optimization parameter [5]. The model has been employed successfully in the design of new ultra-high strength stainless steel grades [6], also considering cost [7], and for corrosion resistant creep steel grades [8]. A similar approach utilizing mesh adaptive direct search (MADS) incorporated in the FactSage system [9] has been developed by Gheribi and Pelton *et al.*, which may be applied to calculate liquid surfaces [10,11] as well as optimizing the composition of magnesium alloys [12] and the blast furnace process [13]. These approaches in the field of materials science preceded the newly started initiative by the U.S. government, the Materials Genome Initiative [14], aimed to accelerate the development of new materials by the development of process-structure-properties based models. Such model-based developments will lead to significant reductions in material development costs.

In this paper efforts have been made to expend the genetic alloy design concept and model the steel making process, in particular the vacuum degassing part. The vacuum degassing has been studied earlier by combining computational fluid dynamics (CFD) and thermodynamics [15,16,17]. In these studies the process was analyzed and a detailed understanding was gained, e.g., on how individual process parameters influence the vacuum degassing process. However, no attempt was made to optimize the vacuum degassing systemically. The present work is a first step to implement the genetic alloy design approach [5] on the steel making process. More specifically, self-consistent CALPHAD-type thermodynamic multi-component databases and empirical kinetic relations are coupled with genetic algorithms to predict the optimal set-up for ladle vacuum degassing. Since this work focus on demonstrating how genetic algorithms can be used to optimize the steelmaking process it should be emphasized that less effort has been spent on the physical and kinetic models. This could affect the results, but will still demonstrate the effectiveness of the methodology presented.

The steel plant’s objective is to efficiently produce high quality steels. Profit is made if the cost can be reduced while strictly fulfilling the demands for a specific steel grade. One way to reduce the cost of the steel making process is to minimize the time needed for one of the most expensive process steps, namely the vacuum degassing which is part of secondary metallurgy. It is performed after deoxidation and alloying, and involves low pressure and high stirring rates. It has been shown to be a very efficient way to reduce the sulfur, nitrogen and hydrogen content in liquid steel [18,19], and to reduce the inclusion content. More information regarding vacuum degassing is found in [18,19] amongst others.

## 2. Model Description

**Figure 1:**
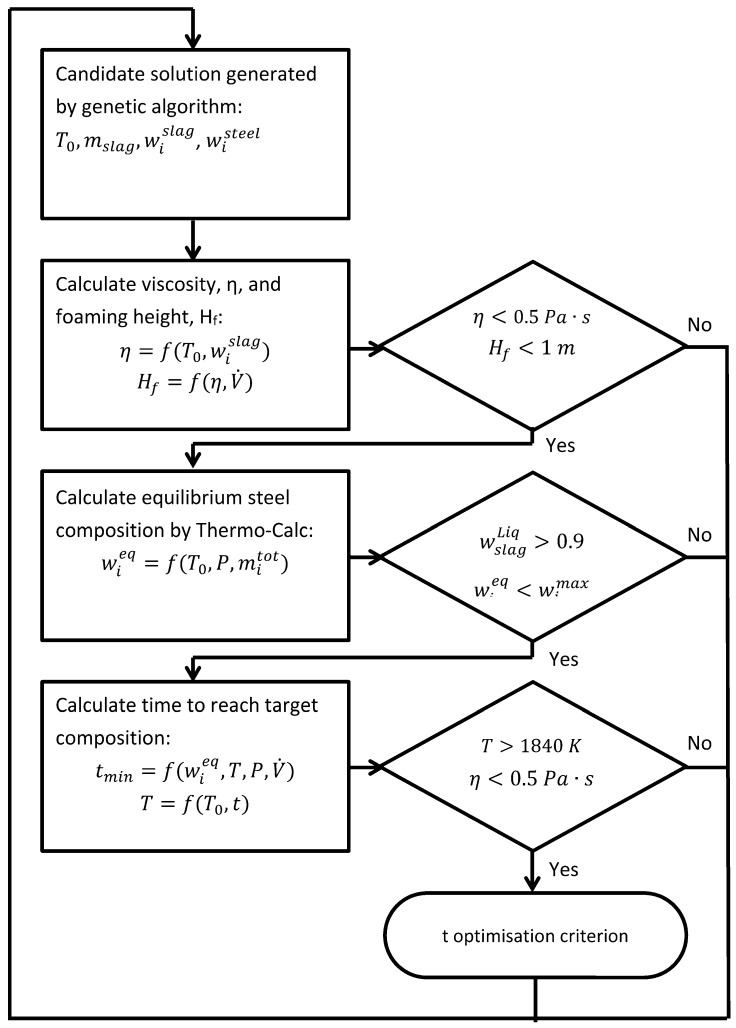
Flow chart of a model for optimizing the minimum time to reach target composition during vacuum degassing.

In this study, the only optimization objective is the degassing time. A simple GA model with one fitness function is used,* i.e.*, degassing time, to search for the best solution. In order to optimize the process it is necessary to have a model that describes the ladle vacuum degassing for all possible set-ups. Since the objective of the present work is to minimize the time, $\;t_{\text{min}}$ , needed to reach target composition, the model should return this value for a certain set-up,* i.e.*, as given by Equation (1):

(1) $$t_{\text{min}}=\text{ degassing}(v_{1},v_{2},\ldots )$$

where $v_{i}$ denotes variables of a set-up generated by the optimization algorithm. The equilibrium information is obtained using the thermodynamic software Thermo-Calc [20], as described in Section 2.1, and the time is obtained using an empirical relationship for the mass transfer, as described in Section 2.2. It should be mentioned that not all solutions explored during the genetic algorithm search are meaningful from a metallurgical perspective. In other words, solutions that violate the physical constraints for vacuum degassing are assigned a very low fitness value and hence practically discarded in producing the next generations. The following constraints are considered in the present work: too high a slag viscosity, too high a foaming height, too low a fraction of liquid slag, and too high a temperature drop. The flow chart of the model for the process-time optimization using the GA approach is given in Figure 1. For each candidate solution the first step is to calculate and confirm that the viscosity and foaming height are within the allowed ranges. The next step is to calculate the thermodynamic equilibrium for the set-up. Using the equilibrium information, it is possible to check that the amount of liquid slag is high enough and that the equilibrium content of O, S, N, Al and Si is below the critical composition. The final step is to calculate the time needed to reach the target composition of O, S, N, Al and Si but also to check that the temperature remains high enough. Detailed descriptions of all the steps indicated in the flow chart are given in the following sections.

### 2.1. Thermodynamic Calculations

Thermodynamic calculations are performed using software, such as Thermo-Calc, together with self-consistent thermodynamic databases for multicomponent alloy systems. Such databases have been developed using the so-called CALPHAD approach, see [1,21,22]. The idea of the CALPHAD approach is to determine Gibbs energy expressions for each individual phase as a function of constitution, temperature and pressure. The determination is performed through an optimization procedure in which model parameters are fitted to experimental, as well as ab initio information. Thermodynamic models based on the crystallography are assigned to each phase, and the majority of models used are found within the Compound Energy Formalism [23]. CALPHAD-type thermodynamic databases have predictive power if all-important binary and most ternary sub-systems have been described. It may then reasonably well predict properties such as phase composition, phase fractions and thermochemical properties such as heat capacity. In the present case the equilibrium state is calculated by considering all elements both in steel and slag to obtain the fraction of impurities in the steel as well as the fraction of liquid slag. 

All thermodynamic calculations are performed with the commercial software Thermo-Calc [20] using a custom-made database using the liquid steel phase as well as solid oxide and sulfide phases from the steel database TCFE6 [24], the liquid slag phase from the database SLAG3 [25] and the gas phase from the database SSUB4 [26]. The custom-made database contains a description of the chemical system Fe–Al–Ca–Mg–Mn–N–O–S–Si.

Although in reality the system is not expected to reach full equilibrium during vacuum degassing the thermodynamic description will provide vital information concerning elemental distribution between the steel and the slag. In many cases the system may be considered to be reasonably close to equilibrium after vacuum degassing, which can be seen as reduced mass transfer in the earlier work [15]. During vacuum degassing the desulfurization is the major reaction that occurs between steel and slag. In the desulfurization the reaction between S, O and Ca dominates. However, Al and Si are also of importance since they react with oxygen that is dissolved in the liquid steel as a result of the desulfurization. Another important reaction during vacuum degassing is the metal-gas reaction through the assembling of N and H from the steel into the gas. In this work only nitrogen is taken into consideration since the current thermodynamic databases do not include hydrogen. Carbon will also interact with N due to the formation of CO. However, due to uncertainties regarding kinetic relations over the various gas species carbon is left out from the calculation. By assuming this, the result is only reliable for steels with low carbon content. The lining of the ladle walls consists of MgO, which may dissolve into the slag depending on the MgO activity. Even though some MgO is expected to be dissolved during vacuum degassing, the effect on the overall equilibrium is likely to be limited since this reaction mostly occurs before the vacuum degassing. The mass fraction of liquid slag is important to take into consideration since a low fraction of liquid slag results in a higher effective viscosity aswell as in a lower mass transfer rate. The amount of liquid slag is obtained from the thermodynamic calculation. 

### 2.2. Kinetic Considerations

The kinetics of the steel making process is complex and involves phenomena such as turbulent flow and heat gradients. This means that the kinetics is more or less unique for every steel plant. Several studies have been conducted to investigate these phenomena in detail, see e.g., [16]. Unfortunately, these complex models need too long computational times to be coupled with genetic algorithms. Moreover the aim of this work is to develop a method to optimize the process over a wide range of various set-ups rather than describing one particular set-up in detail. However, in order to be able to control the process at the steel plants, simpler analytical process models may be used [16]. Although the exact nature of these models is considered trade secrets it is possible to use a similar approach by assuming reasonable kinetic relationships. The mass fraction of element *i* in the steel at time *t*, $w_{i,\text{steel}}(t)$, may be calculated using Equation (2):

(2) $$w_{i,\text{steel}}(t)=\left(w_{i,\text{steel}}^{0}-w_{i,\text{steel}}^{\text{eq}}\right){\text{e}}^{-k_{i}t}+w_{i,\text{steel}}^{\text{eq}}$$

where $w_{i,\text{steel}}^{0}$ and $w_{i,\text{steel}}^{\text{eq}}$ denotes the initial and equilibrium mass fraction of element *i* in the steel, respectively, and $k_{i}$ the mass transfer coefficient of element *i*. The reaction rate decreases exponentially with time since the driving force decreases as the reaction progresses. If instead the objective is to calculate the time needed to reach a specific composition, $w_{i,\text{steel}}^{\text{max}}$ , this is done by setting $w_{i,\text{steel}}\left(t\right)=w_{i,\text{steel}}^{\text{max}}$ and rearranging the equation into Equation (3). Of course, both $w_{i,\text{steel}}^{\text{eq}}$ and $k_{i}$ depend on external conditions, such as temperature. Constant values of these parameters are used for simplification. These values are calculated 20 K below the start temperature, which yields an average value, assuming a 20 min process time and temperature drop of 2 K/min. Since the process time of a good solution is expected to be less than 20 min this approximation will not generate too optimistic times. Using water models it has been shown that the mass transfer coefficient strongly depends on the stirring energy, $\dot{\text{ε }}$, according to Equation (4) [27]:

(3) $$t=-\frac{1}{k_{i}}\text{ln}\frac{w_{i,\text{steel}}^{\text{max}}-w_{i,\text{steel}}^{\text{eq}}}{w_{i,\text{steel}}^{0}-w_{i,\text{steel}}^{\text{eq}}}$$

(4) $$k_{i}=C{\dot{\text{ε }}}^{n}$$

where *C* is a constant and *n* the exponential factor, which from experimental practice is known to be 2.1, if the stirring energy is above 60 W/ton, and 0.25, if the stirring energy is lower [18,19]. The reason for the abrupt change around 60 W/ton is considered to be the result of slag and steel mixing at the higher stirring energies while at lower stirring energies only the steel is circulated [19]. In the latter case there is much less steel and slag interaction. In the current paper *C* is estimated to be around $2.7\times 10^{-8}\;$ s^−1^ for all elements involved in slag and steel reactions, using results from CFD calculations [16]. These calculations were performed in order to develop an on-line process model for vacuum degassing. Since the nitrogen removal does not rely on the steel and slag reaction, but rather on the steel and gas reaction, it does not follow the same relation. However, lacking a better description of the nitrogen transfer, and since nitrogen removal has approximately the same order of magnitude as desulfurization, it is assumed to have the same value. The assumption that nitrogen would behave like sulfur is unphysical but will demonstrate how the equilibria involving different impurity elements will impact the minimum time needed to reach the target composition. In this work the stirring energy, $\dot{\varepsilon }$, is calculated using an empirical relation [18], as shown in Equation (5), that have been derived from [28]:

(5) $$\dot{\text{ε }}=\;14200\frac{\dot{V}T}{m_{\text{steel}}}\text{log}\left(\frac{1+h}{1.48P}\right)$$

where $\dot{V}$ denotes the gas flow rate (m^3^/min), *T* the temperature (K) , *m*_steel_ the steel mass (kg), *h* the depth of the steel bath (m), and *P* the pressure (Pa).

### 2.3. Constraints

Even though the effect of physical properties on the mass transfer rate is neglected in the present optimization model, each candidate solution generated by genetic algorithm still has to fulfill some physical constraints. The constraints considered here are the slag viscosity and foaming height. In addition, the temperature drop will change the equilibrium steel composition and thus dynamically change the mass transfer coefficient. The effect of the temperature change during the degassing process is neglected but the temperature drop is checked so that the process does not end up at unreasonable low temperatures.

#### 2.3.1. Viscosity

The viscosity will influence how efficient the vacuum degassing will be. A high viscosity is expected to have a negative influence on the reaction rate. The viscosity is not included in the kinetic relationship. Instead a constraint is used which does not allow the viscosity to be above 0.5 Pa·s. Although this assumption might generate an incorrect minimum time it discards solutions that are too slow due to high viscosity. The viscosity, η, is calculated using the Urbain model [29]. In the Urbain model the viscosity is obtained from Weymann type equation [30] as is seen in Equation (6):

(6) $$\text{η}=a_{1}T\text{exp}\left(\frac{1000a_{2}}{T}\right)[\text{Pa}\cdot \text{s}]$$

where *T* denotes the temperature and *a*_1_ and *a*_2_ are composition dependent parameters, see [29,31] for details about *a*_1_ and *a*_2_.

#### 2.3.2. Foaming Height

If the steel is subjected to a gas flow or gas formation, slag foaming will occur. In some cases, like in an electric arc furnace, this is desirable because the foam acts as thermal insulation. In vacuum degassing, however, slag foaming is undesirable since too high a slag rise may cause damage to the equipment. The foaming height, $H_{\text{f}}$, is calculated by using Equation (7) derived from [32]:

(7) $$H_{\text{f}}=1150\frac{\text{η}}{\sqrt{\text{σρ}}}\frac{\dot{V}}{A}\left[\text{m}\right]$$

where η denotes the viscosity (Pa·s), σ the surface tension (N/m), ρ the density of the slag (kg/m3), $\dot{V}$ the volumetric flow rate (m^3^/min), and the *A* the area of the steel bath (m^2^).

#### 2.3.3. Temperature Drop

The temperature drop is mainly due to heat transfer from the steel to the ladle walls. Some heat will be lost by heating the slag, which is colder than the steel. However, this neglected since the steel amount is much higher than slag and also because the slag foaming. Which is expected to be higher during vacuum degassing and this will reduce the heat loss through the slag. It is easily understood that the temperature drop will be more severe with increased stirring. Due to the high stirring energy during vacuum degassing the temperature drop is expected to be higher than during other ladle operations. It is assumed that the temperature decrease due to stirring may be described as $C{\dot{\text{ε }}}^{n}$, just like the mass transfer, since stirring will make new steel come into contact with the ladle wall or slag continuously. Given the similarity *n* is set to 0.25 which is the relationship between the mass transfer coefficient and the stirring energy when no mixing between slag and steel occurs [19]. Hence, the temperature drop is estimated using the following Equation (8):

(8) $$\frac{\text{d}T}{\text{d}t}=-1-0.25{\dot{\text{ε }}}^{0.25}[\text{K}/\text{min}]$$

where the constants in the equation are obtained by assuming a temperature drop of about 1 K/min under normal ladle operations (assuming atmospheric pressure and no or very low gas flow) and about 2 K/min during vacuum degassing (assuming 50 Pa pressure and 0.2 m^3^/min gas flow). Details about the estimation of the temperature drop can be found in [19].

## 3. Parameter Settings

In order to minimize the process time needed to reach the target composition both the steel and slag compositions should be optimized such that the driving force for reducing impurities is as favorable as possible. The eight variables used as optimizing variables for degassing are listed in Table 1. The values result from the operations performed before degassing while pressure and flow rate are process parameters for the degassing process. The two major process parameters during vacuum degassing are flow rate and pressure. Using the current kinetic relationship the flow rate will only affect the time needed to reach the target composition and not the optimal set-up. The pressure will have a similar effect as the flow rate, meaning that lower pressures will result in shorter times. In addition, the pressure also has an effect on the equilibrium. The optimizations are performed at four different pressures (50, 75, 100 and 200 Pa).

**Table 1:** Ranges for all variables used in the optimization.

Input	Al	Ca	Si	Fe	Slag	Al_2_O_3_	MgO	SiO_2_	CaO	Temperature
	(wt%)	(wt%)	(wt%)	(wt%)	(kg)	(wt%)	(wt%)	(wt%)	(wt%)	(K)
min	0.0	0.0	0.0	Balance	1000	20	5	5	Balance	1913
max	0.04	0.005	0.3	–	2000	40	10	20	–	1973

The initial composition of the steel is fixed to 1.5 wt% Mn, 10 ppm O, 50 ppm N and 200 ppm S. Additionally a fixed amount of 20 kg FeO is added to the slag and the amount of steel per heat, *m*_steel_, is set to 10^5^ kg. The depth of the steel bath, *h*, is set to 2 m, the upper surface area, *A*, is set to 7 m^2^, the density of the slag, ρ, is set to 3000 kg/m^3^ and the flow rate, $\dot{V}$, is set to 0.2 m^3^/min. These values are needed to calculate the stirring energy, $\dot{\varepsilon }$, and thus the time needed to reach the critical composition. The surface tension of the slag, σ, varies with composition and temperature but should be above 0.4 N/m, for a slag consisting mostly Al_2_O_3_–CaO–SiO_2_ and with 20 mass% SiO_2_ in the temperature range 1800–2000 K [33]. Given that the surface tension does not exceed this value and that the viscosity condition is fulfilled the foaming height will not exceed 0.15 cm. The degassing is considered as completed when the conditions listed in Table 2 have been reached. O, S and N should be below the critical value as they are considered as impurities. For many steel standards there is lower limit for Si but for simplicity only an upper limit is set for Si and Al.

**Table 2:** Target composition of steel after vacuum degassing.

Element	S (ppm)	N (ppm)	O (ppm)	Al (wt%)	Si (wt%)
max	100	30	5	0.02	0.25

The temperature used in the thermodynamic calculations as well as the calculation of the viscosity and the mass transfer, is set to 20 K below the temperature generated by the genetic algorithm in order to take temperature drop into consideration. The final temperature is used to check that the viscosity is below the critical value when the degassing has finished. The temperature is not allowed to drop below 1840 K and the viscosity should never be allowed to reach above 0.5 Pa·s and the foaming height should not be above 1 m.

The optimization is performed using genetic algorithms, which mimics evolutionary processes in natural systems, following the survival of the fittest principle. The heuristic evolution is controlled by probabilistic operators, such as selection, crossover, and mutation rather than deterministic functions. Each candidate solution is coded as a binary string (chromosome) by concatenating the concentration of each element expressed in base-2 (genes). 3 binary bits are linked to each component wherein 000 stands for the minimum concentration and 111 refers to the upper boundary as given in Table 1. In this way, there are 2^3^ = 8 candidate concentrations for each component distributed equally between the concentration limits of each element and represented by 3 bits. Thus, each potential composition set is represented by a binary string of 3 × 8 = 24 bits, which is a good trade-off between precision and computational time. It means the step is in the range of the precision of the variables that can be expected under normal operation. It is also low enough so that every optimal set-up was generated in less than 10,000 iterations. This could be compared to more than 16 million iterations that would be necessary if every possible solution was to be calculated. In fact the solution reaches the optimum well before 10,000 iterations, however, sufficient extra calculations were still performed to confirm that no better solution can be found and hence the convergence to the global optimum. In fact the solution reaches the optimum well before 10,000 iterations, however, sufficient extra calculations were still performed to confirm that no better solution can be found and hence the convergence to the global optimum. This global optimum is further confirmed by running independent calculations starting from different random initial solutions while reaching the same optimum regardless their different optimization passes. More details on the optimization procedure can be found elsewhere [5] as well as details about the genetic algorithm program used [34].

## 4. Results and Discussion

All the results from the optimizations are listed in Table 3 together with normal values used by industry. One interesting feature is that the optimal set-up seems to depend highly on the pressure. With increasing of the pressure the content of Al in the steel, the SiO_2_ in the slag and the temperature increase monotonically, while the opposite behavior is seen for Al_2_O_3_. The result obtained at 50 Pa pressure is within the range expected from industrial practice with two major exceptions, the slag amount and the SiO_2_ content. The difference in slag amount, however, is expected since it is known that a higher slag content promotes desulfurization, see, e.g., a CFD study of vacuum degassing [17]. In a steel plant the amount of SiO_2_ is expected to be relatively high since some SiO_2_ remains from earlier production stages as well as from the Si-deoxidation. The fact that industrial practice suggests higher pressure indicates that the model used cannot fully reproduce the true nature of vacuum degassing. One likely reason is that the transfer coefficient of nitrogen may be under-estimated. However, the result obtained at 50 Pa can be used to demonstrate the methodology.

**Table 3:** Results from the optimizations. Values according to industrial practice are shown in the bottom row.

Pressure (Pa)	Time (s)	Al (wt%)	Ca (wt%)	Si (wt%)	Slag (kg)	Al_2_O_3_ (wt%)	MgO (wt%)	SiO_2_ (wt%)	Temperature (K)
50	429	0.010	0.005	0.243	1571	25.7	7.14	5.00	1922
75	479	0.019	0.005	0.243	1143	22.9	7.86	7.14	1964
100	562	0.023	0.005	0.243	1000	20.0	9.29	11.43	1973
200	1002	0.036	0.005	0.243	1286	20.0	8.57	15.71	1973
100	900–1200 ^†^	0.020–0.050	0–0.0010	0.20–0.30	1000	25.0–30.0	5.0–10.0	8.0–12.0	1950

^†^ Indicates normal process time,* i.e.*, longer than the minimum time.

**Figure 2:**
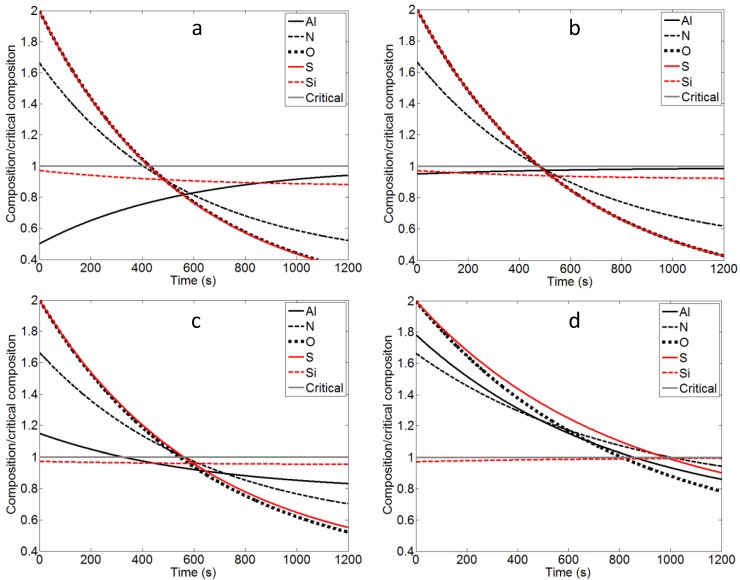
Composition of the Al, N, O, S and Si normalized to critical level *vs*. time. Calculated for solutions obtained at pressures of (**a**) 50 Pa; (**b**) 75 Pa; (**c**) 100 Pa and (**d**) 200 Pa.

To investigate the evolution of concentrations of critical alloying elements during degassing, their variation with time at various pressures are calculated and plotted in Figure 2. In all cases the Si content is below the critical composition during the whole process stage. At lower pressures (50 and 75 Pa) the Al content is below the critical content from the beginning while this is not the case at higher pressures (100 and 200 Pa). Al is not limiting the optimal time in any of the optimal set-ups. In all cases, particularly at lower pressures, the content of S and O almost overlap. The reason for this is that the equilibrium content of both O and S is only a small fraction of their maximum allowed content. Interestingly, the positions of the cross points of N and S change with pressures giving rise to the change of the dominant factor in optimal degassing time. At the lowest pressure (50 Pa), the intersection is below the critical line. Therefore, it takes more time for the concentration of S to reach its critical level, indicating that the removal of S is the determining factor. At pressures of 75–100 Pa, the intersections are located right on the critical line. Thus N and S removal lead to the same minimal degassing time. Finally, at the higher pressure (200 Pa) the intersections of N and S is above the critical line and thus N becomes the determining factor for the degassing time. These trends are expected since low pressures promote nitrogen removal both via thermodynamics and by increased stirring while desulfurization is only affected by the stirring. It should be stated that irrespective of the pressure level, the positions of cross point are always close to the critical line indicating that the system is generally tailored such that N and S nearly simultaneously reach the intended minimal values.

**Figure 3:**
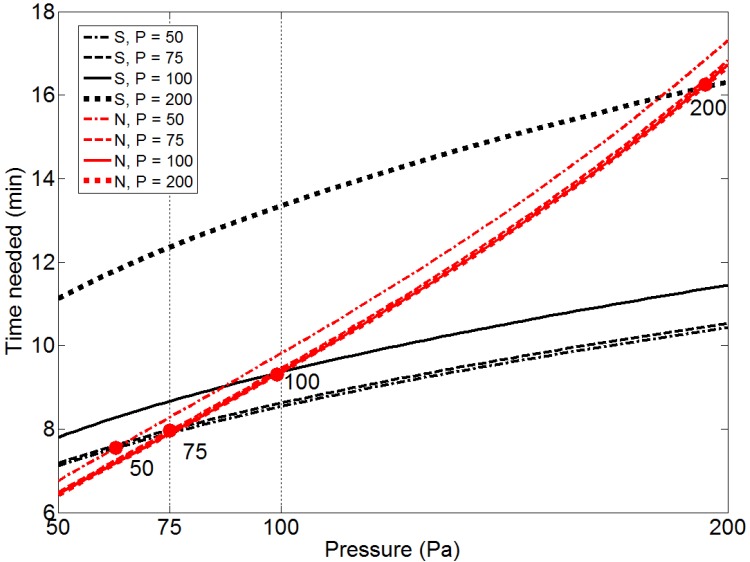
Time needed to reach target composition of S and N as function of pressure, using optimal set-ups found at *P* = 50, 75, 100 and 200 Pa. The circles indicate the pressure above which the time to reach the nitrogen composition is limiting.

Since different pressures yield considerably different optimal set-ups, the optimized process time at various pressures and corresponding set-ups is analyzed in more detail and the results are shown in Figure 3. In the figure the times needed to reach both the maximum allowed content of S and N are compared. Again it can be seen that the sulfur level is the limiting factor at lower pressures. It is interesting to note that the pressure at which the critical content of N and S are reached simultaneously is close to the pressure where the corresponding optimal set-up is found. This again demonstrates how the optimal set-up takes both desulfurization and nitrogen removal into consideration. However, the optimal set-up obtained at 200 Pa is only slightly better considering nitrogen removal, while it is disadvantageous considering desulfurization as the pressure decreases. 

**Figure 4:**
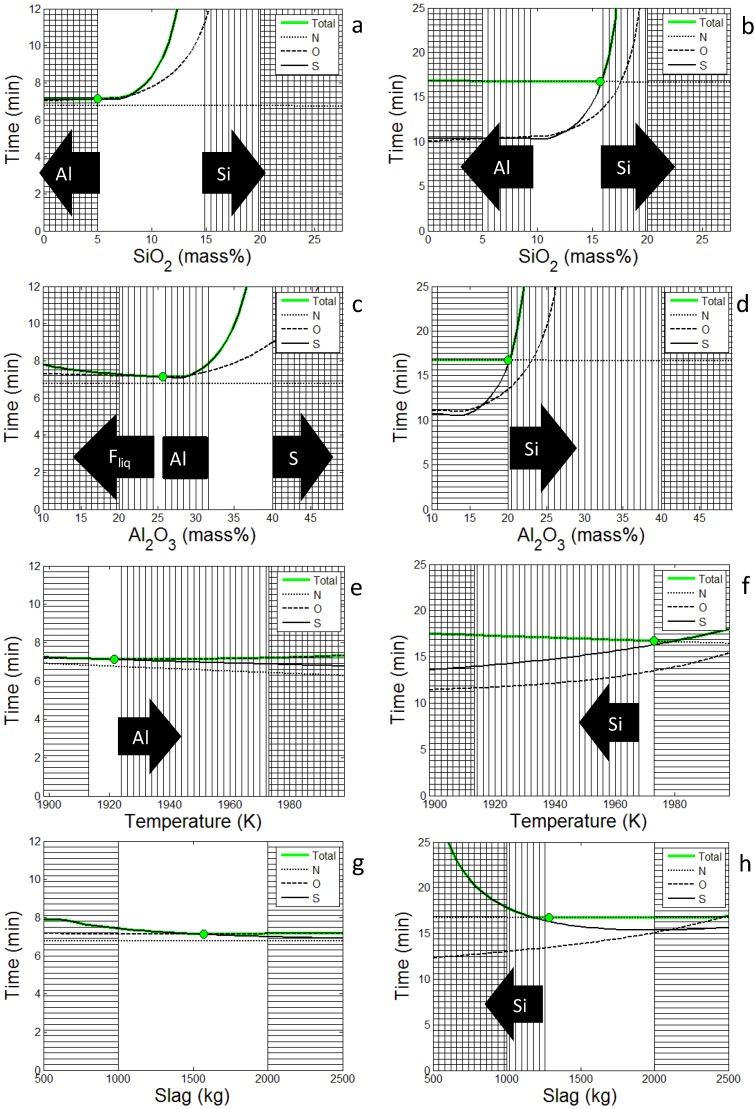
Time to reach target composition in total, N, S and O plotted against optimization variables, SiO_2_ (**a**,**b**), Al_2_O_3_ (**c**,**d**), initial temperature (**e**,**f**) and slag amount (**g**,**h**). All other optimization variables are fixed to the optimal set-up. The left side (**a**,**c**,**e**,**g**) is using the solution at 50 Pa and the right side (**b**,**d**,**f**,**h**) is the solution at 200 Pa. Vertical lines means that some criterion has not been met and horizontal lines means that the variable is outside the search domain. The arrows show which constraint that is broken.

For all solutions the optimal amounts of the refining elements in the steel (Al, Ca and Si) reach the highest possible value, according to the constraints. The optimal solution obtained at 50 Pa indicates that some Al is dissolved from the slag into the steel, while the opposite is seen at higher pressures. In Figure 4 the optimal set-ups found at 50 Pa and 200 Pa are varied with respect to some critical variables. As can be seen in Figure 4a,b desulfurization is most effective if the SiO_2_ content is below a certain value while the nitrogen removal is more or less unaffected. However at high pressure the nitrogen removal is limiting which indicates that the SiO_2_ content will be optimized with respect to the nitrogen removal as seen in Figure 4b. Al_2_O_3_ on the other hand has one optimal value, which strongly depends on the remaining parameters as shown in Figure 4c,d, again the nitrogen removal is limiting at higher pressures. The time needed to reach the critical content of nitrogen, sulfur and oxygen in the steel depends on temperature as shown in Figure 4e,f. However, while nitrogen removal simply is enhanced with increasing temperature, the effect on sulfur and oxygen depends on other parameters as well. Thus, since different pressures yield different optimal set-ups, particularly regarding the slag composition, the temperature effect is different for sulfur and oxygen. In Figure 4g,h the positive effect of increasing the slag amount on desulfurization is clearly demonstrated. However, using the optimal set-up found at 200 Pa the time needed to reach the critical oxygen content increases with the slag amount which has a negative effect on the desulfurization as seen at higher pressures in Figure 4h. In general, it is shown that the nitrogen removal becomes limiting as the pressure increases.

The fact that the optimal set-up at low pressures is close to industrial practice shows that the methodology can reproduce what is known to be good slag and steel compositions. The fact the agreement with industrial practice is observed at lower pressures than expected indicated that the current description of nitrogen transfer should be improved. Using this approach it should be possible to predict a good slag composition as well as the amount of refining elements needed to produce a certain steel grade. In principle the process optimization approach as described in this paper, which originally was developed for alloy design, could be extended and applied on other process steps as well, e.g., decarburization of stainless steel, melting of scrap in an electric arc furnace or even on multi-stage processes. It could be used to design of new processes and materials but also calibration of existing processes.

## 5. Conclusions

By using a materials by design approach in which a genetic algorithm, computational thermodynamics and analytical process models are combined, a model has been developed to minimize the vacuum degassing time as a function of the steel and slag compositions. Despite the simple analytical description of the mass transport kinetics, the predicted optima are close to industrial practice. Lower pressure than expected from industrial practice is required to generate the optimal set-up, which is a result of the simple relationship for the nitrogen mass transfer. For the optimal set-up the target contents of sulfur, nitrogen and oxygen are reached almost simultaneously. The predicted optimal slag amount is larger than used in normal industrial operation, however, it has previously been established that increased slag amount is beneficial for desulfurization. At lower pressures the optimal set-up is stable; in most cases the process time decreases until the variable hits one of the physical constraints. At higher pressures there are some cases where the optimal results are almost unaffected by a change of a variable, the reason being that the nitrogen content becomes limiting. 

## References

[1] Kaufman L., Bernstein H. (1970). Computer Calculation of Phase Diagrams.

[2] Spencer P.J. (2008). A brief history of CALPHAD. Calphad.

[3] Campbell C.E., Olson G.B. (2001). Systems design of high performance stainless steels I. Conceptual and computational design. J. Comput.-Aided Mater. Des..

[4] Olson G.B. (1997). Computational design of hierarchically structured materials. Science.

[5] Xu W., Rivera-Díaz-del-Castillo P.E.J., van der Zwaag S. (2008). Genetic alloy design based on thermodynamics and kinetics. Philos. Mag..

[6] Xu W., Rivera-Díaz-del-Castillo P.E.J., Wang W., Yang K., Bliznuk V., Kestens L.A.I., van der Zwaag S. (2010). Genetic design and characterization of novel ultra-high-strength stainless steels strengthened by Ni_3_Ti intermetallic nanoprecipitates. Acta Mater..

[7] Xu W., van der Zwaag S. (2011). Property and cost optimisation of novel UHS stainless steels via a genetic alloy design approach. ISIJ Int..

[8] Lu Q., Xu W., van der Zwaag S. (2013). Computational design of precipitation strengthened austenitic heat-resistant steels. Philos. Mag..

[9] The FactSage System. http://www.factsage.com/.

[10] Gheribi A.E., Audet C., le Digabel S., Bélisle E., Bale C.W., Pelton A.D. (2012). Calculating optimal conditions for alloy and process design using thermodynamic and property databases, the FactSage software and the Mesh Adaptive Direct Search algorithm. Calphad.

[11] Gheribi A.E., Robelin C., le Digabel S., Audet C., Pelton A.D. (2011). Calculating all local minima on liquidus surfaces using the FactSage software and databases and the Mesh Adaptive Direct Search algorithm. J. Chem. Thermodyn..

[12] Gheribi A.E., le Digabel S., Audet C., Chartrand P. (2013). Identifying optimal conditions for magnesium based alloy design using the Mesh Adaptive Direct Search algorithm. Thermochim. Acta.

[13] Harvey J.-P., Gheribi A.E. (2014). Process simulation and control optimization of a blast furnace using classical thermodynamics combined to a direct search algorithm. Metall. Mater. Trans. B.

[14] (2011). Materials Genome Initiative for Global Competitiveness.

[15] Jonsson L., Sichen D., Jönsson P. (1998). A new approach to model sulphur refining in a gas-stirred ladle: A coupled CFD and thermodynamic model. ISIJ Int..

[16] Hallberg M., Jonsson T.L.I., Jönsson P.G. (2004). A new approach to using modelling for on-line prediction of sulphur and hydrogen removal during ladle refining. ISIJ Int..

[17] Hallberg M., Jonsson L.T.I., Jönsson P.G., Undvall P. (2005). Sulphur and hydrogen refining during vacuum degassing: A new concept for process control. Stahl Und Eisen.

[18] Kor G.J.W., Glaws P.C., Fruehan R.J. (1998). Ladle refining and vacuum degassing. The Making, Shaping and Treating of Steel.

[19] Szekely J., Carlsson G., Helle L. (1989). The fundamental aspects of injection metallurgy. Ladle Metallurgy.

[20] Andersson J.-O., Helander T., Höglund L., Shi P., Sundman B. (2002). Thermo-Calc & DICTRA, computational tools for materials science. Calphad.

[21] Saunders N., Miodownik A.P. (1998). CALPHAD (Calculation of Phase Diagrams): A Comprehensive Guide.

[22] Lukas H., Fries S.G., Sundman B. (2007). Computational Thermodynamics: The Calphad Method.

[23] Hillert M. (2001). The compound energy formalism. J. Alloys Compd..

[24] (2009). TCFE6–TCS Steels/Fe-Alloys Database v6.2. Thermo-Calc Software AB. http://www.thermo-calc.com/.

[25] (2010). SLAG3–TCS Fe-containing Slag Database v3.1. Thermo-Calc Software AB. http://www.thermo-calc.com/.

[26] (2008). SSUB4–SGTE Substances Database v4.1. Thermo-Calc Software AB. http://www.thermo-calc.com/.

[27] Asai S., Kawachi M., Muchi I., Carlsson G. (1983). Mass transfer rate in ladle refining processes. SCANINJECT III, Refining of Iron and Steel by Powder Injection.

[28] Pluschkell W (1981). Grundoperation pfannenmetallurgischer Prozesse. Stahl Und Eisen.

[29] Urbain G., Cambier F., Deletter M., Anseau M.R. (1981). Viscosity of silicate melts. Trans. J. Br. Ceram. Soc..

[30] Weymann H.D. (1962). On the hole theory of viscosity, compressibility, and expansivity of liquids. Kolliod-Zeitschrift Zeitschrift Polym..

[31] Mills K.C., Eisenhüttenleute V.D. (1995). Viscosities of molten slags. Slag Atlas.

[32] Jiang R., Fruehan R.J. (1991). Slag foaming in bath smelting. Metall. Trans. B.

[33] Keene B.J., Eisenhüttenleute V.D. (1995). Surface tension of slag systems. Slag Atlas.

[34] Carroll D.L. (2001). FORTRAN Genetic Algorithm (GA) Driver v 1.7a. http://www.cuaerospace.com/Technology/GeneticAlgorithm/GADriverFreeVersion.aspx.

